# Cost-effectiveness analysis of the digital fall preventive intervention Safe Step among community-dwelling older people aged 70 and older

**DOI:** 10.1007/s10433-024-00828-8

**Published:** 2024-10-26

**Authors:** Saranda Bajraktari, Marlene Sandlund, Beatrice Pettersson, Erik Rosendahl, Magnus Zingmark

**Affiliations:** 1 Department of Community Medicine and Rehabilitation, Physiotherapy, Umeå University, Umeå, Sweden; 2 Municipality of Östersund, Health and Social Care Administration, Östersund, Sweden

**Keywords:** Accidental falls, Balance and strength exercise, Cost-effectiveness, Digital health, mHealth, Reach

## Abstract

Falls are the most common cause of injury in older people, with consequences for the individual and society. With an increasing population of older people, falls and related costs are expected to increase. It is crucial to identify scalable and cost-effective interventions and subsequently reduce fall-related costs. The aim was to evaluate the cost-effectiveness of the Safe Step digital fall preventive exercise intervention over a period of 12 years and, in addition, to evaluate the impact of increased recruitment cost and decreased intervention effect. The intervention was evaluated in an observational study in a municipality context targeting community-dwelling older people of age 70 + . A Markov model with five states was used to model the cost-effectiveness of the Safe Step intervention and evaluate quality-adjusted life years (QALYs) and fall-related costs from a societal perspective. By using data from a meta-analysis as basis for the estimated intervention effect, the Safe Step intervention was compared with a no-intervention alternative. The results showed that the Safe Step intervention dominated no intervention. In the sensitivity analysis with the most conservative estimate of intervention effect, the ICER was €7 616 per QALY gained. Hence, Safe Step showed to be a cost-saving fall preventive intervention in older people at risk of falling and potentially cost-effective even with a low estimated intervention effect. Future studies on efficacy of fall preventive digital interventions will contribute in precising effect estimates and enhance the validity of these cost-effectiveness results.

## Introduction

Approximately one-third of people aged 65 years and older experience one or more falls per year (World Health Organization, 2021). Those who have fallen previously have an increased risk of falling again (Gribbin et al. 2009). Fall-related injuries represent the type of injury leading to the highest number of hospitalizations, visits to emergency departments, and deaths among older people in Sweden (Gribbin et al. 2009). Falls also affect quality of life negatively due to mobility-related disability, fear of falling, and loss of independence (Borgström et al. 2006). Hence, falls in older people represent a major public health challenge in terms of the incidence and related consequences for the individual’s health and subsequent costs for health and social care (Folkhälsomyndigheten 2022).

According to the Swedish National Board of Health and Welfare, fall-related injuries requiring emergency care within outpatient specialised care or inpatient care cost over €1.5 billion in 2020. Out of this amount, approximately €1 billion represented direct healthcare and social services costs for regions and municipalities in Sweden (Socialstyrelsen 2022a). The risk of falls increases with advanced age; therefore, fall-related costs are expected to increase due to larger numbers of older people. It is therefore crucial to work proactively and identify scalable and cost-effective interventions to prevent falls and support the ageing population to maintain a good quality of life (Lindholm et al. 2013, Socialtjänstlag [SFS], 2001).

There is robust evidence that fall preventive interventions can prevent falls in older people. A systematic review suggests that exercise interventions targeting balance and strength represent the most effective strategy in reducing fall rates, fall risk and, to some extent, fall-related injuries in community-dwelling older people (Sherrington et al. 2020). Despite the established evidence on the benefits of fall preventive interventions, the incidence on falls and related injuries remains high. Factors related to implementation can limit the impact of potentially effective interventions. One such factor is limited reach in populations (Glasgow et al. 1999). Therefore, a factor to consider when estimating cost-effectiveness is the resources required to optimize reach in the population, for example, the cost of recruitment. While the strategies used for recruitment can add cost beyond the actual intervention, they can also contribute to increased reach (Bajraktari et al. 2022) and can thereby expand the utility of the intervention on a population level. Reaching a large number of people with traditional exercise programmes, often overseen by professionals and offered in group or individual settings, would be challenging due to budget limitations and staffing challenges. These resources are usually focused on assisting those with significant healthcare needs and supporting their daily functioning, leaving limited capacity for preventive interventions.

Digital technology provides opportunities to tailor and deliver interventions to a large number of individuals and thereby increase access to such interventions. Older people use mobile technology to an increasingly higher degree, and digital solutions are becoming more accessible (Jeffrey I. Cole, 2017). Several digital applications within fall prevention are currently being evaluated (Hamm et al. 2016). One such intervention is Safe Step which is a fully self-managed exercise programme developed in co-creation with older people aged 70 years and older (Pettersson et al. 2020; Sandlund et al. 2016). Digital fall preventive exercise such as the Safe Step application has the potential to significantly reduce fall rates and is promising to increase reach (Delbaere et al. 2021; Bajraktari et al. 2022). Since resources are limited, and the demand for healthcare is increasing, decision-makers must allocate resources efficiently (Folkhälsomyndigheten 2023; Socialstyrelsen 2023). However, despite the established evidence on exercise interventions to prevent falls, few cost-effectiveness studies have been published (Olij et al. 2018; Winser et al. 2020; Davis et al. 2010). Moreover, studies evaluating cost-effectiveness of digital fall prevention exercise are scarce (Ambrens et al. 2022), thereby limiting the knowledge base for decision-makers. The purpose with this study was to: (i) evaluate the cost-effectiveness of the Safe Step intervention in comparison with a no-intervention alternative in a Swedish municipality context; (ii) explore the impact of higher recruitment costs or smaller intervention effect on the cost-effectiveness of the intervention.

## Method

**Fig. 1:**
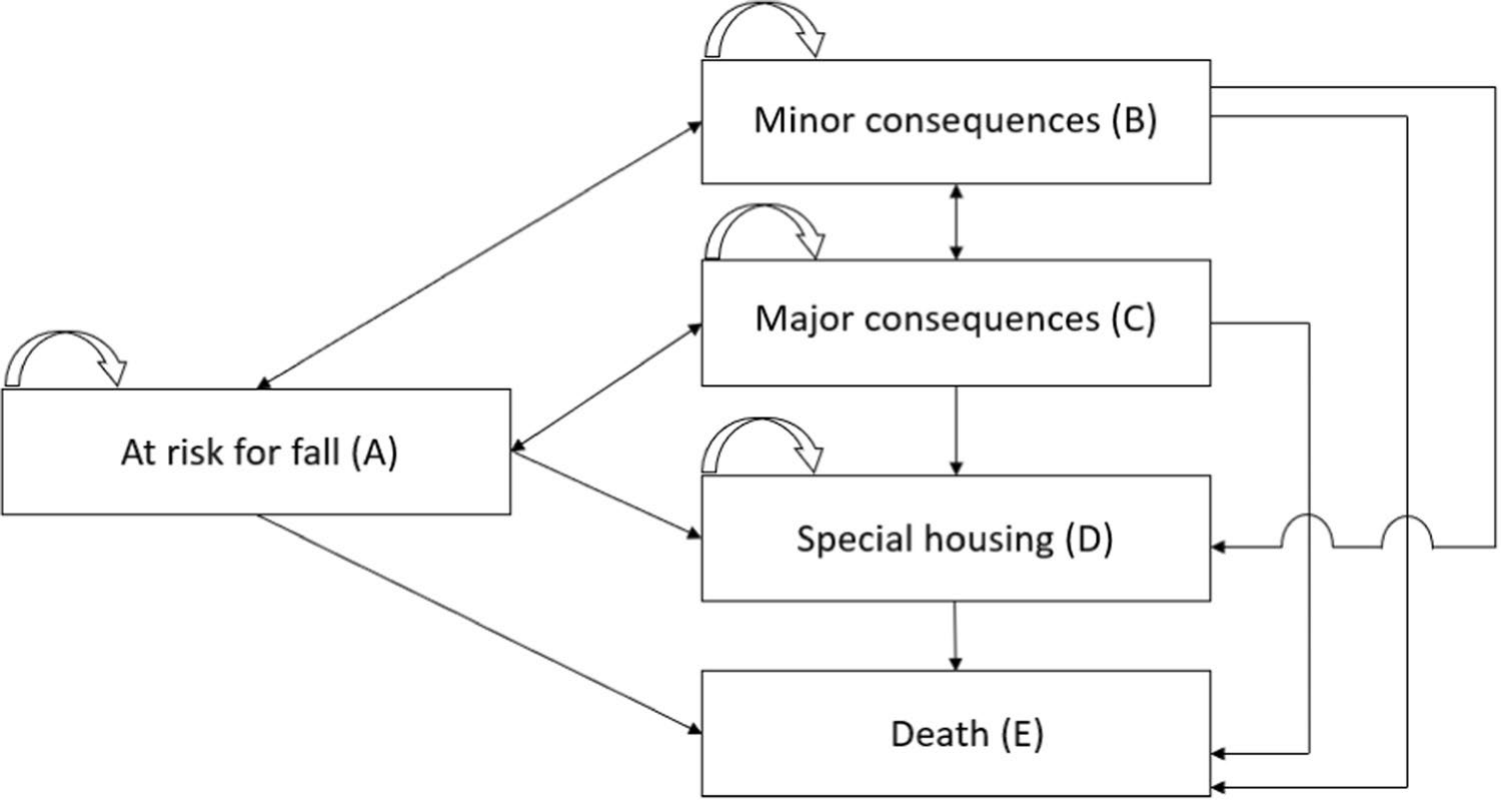
Markov model (states are shown in rectangles, straight arrows represent transition probabilities from one state to the other, curved arrows represent probabilities to remain in the same state). *State A (At risk for fall)* refers to a state where a person either has fallen previously or perceives a decline in balance. A fall in state A does not require any medical attention nor impact daily functioning (such as increased need for informal care or home help). *State B (Minor consequences after a fall) r*efers to a state in which a person has experienced a fall that has led to a visit to the emergency care department without being admitted to hospital. A fall in state B is assumed to have no impact on daily functioning (such as increased need for informal care or home help) but might require follow-up visits for rehabilitation. *State C (Major consequences after a fall)* refers to a state where a person has experienced a fall which has led to an emergency visit and hospital admission. On discharge from the hospital, the person will require rehabilitation; this fall will impact daily functioning (such as increased need for informal care or home help). *State D (Special housing after a fall) r*efers to a state where a person has experienced a fall that has led to need for long-term care in special housing. Persons in state D are assumed to remain in this state over time or transition to *state E* (*Death*)

To address the aim of the study, we developed a Markov model with a time horizon of 12 years including five states representing risk of a fall and consequences of varying severity following a fall (Fig. 1). The Safe Step intervention was evaluated at a population level within the context of a Swedish municipality. Since no other digital fall preventive intervention had been implemented at a population level, Safe Step was compared to no intervention in the analysis. The model parameters included transition probabilities between the states in the model (Table 1), health-related quality of life (QoL) and societal costs for each state (Table 2), as well as intervention costs and intervention effect (Table 3). The study followed the Consolidated Health Economic Evaluation Reporting Standards (CHEERS) statement (Husereau et al. 2022).

**Table 1:** Transition probabilities^a^ for annual transitions between states in the Markov model

	A	B	C	D	E
No intervention					
A	0.72	0.07	0.13	0.05	0.03
B	0.59	0.13	0.20	0.05	0.03
C	0.28	0.09	0.20	0.23	0.20
D	0.00	0.00	0.00	0.60	0.40
E					1.00
Safe Step intervention					
A	0.77	**0.05**	**0.10**	0.05	0.03
B	0.64	0.13	**0.15**	0.05	0.03
C	0.28	0.09	0.20	0.23	0.20
D	0.00	0.00	0.00	0.60	0.40
E					1.00

^a^Transition probabilities in bold apply only to the first year of the intervention period and represent the sole difference between the intervention and no-intervention alternatives

**Table 2:** Estimates of annual costs per person (€) and quality of life (QoL scores)

Markov states	QoL scores	Source	Cost (€)	Source
A	0.86	Bajraktari et al. (2022)	0	–
B	0.72	Borgström et al. (2006)	707	Socialstyrelsen (2022a)
C	0.59	Borgström et al. (2006)	18 625	Socialstyrelsen (2022a)
D	0.41	Zingmark et al. (2016)	99 727	Socialstyrelsen (2022a)
E		–	0	–

**Table 3:** Intervention costs in (€)

Costs included^a^	Intervention costs		
	Units	Total costs	Cost per participant (n = 173)
Recruitment			
Brochure	7500	6707	39
Bus advertisement	64	1600	9
Oral presentations	23	600^b^	4
Supportive strategies			
Group exercise sessions	30	6900	40
Introductory and technical support drop-in meeting	10	256^b^	2
Application maintenance	NA	4581	27
Total		20 644	121

^a^Costs are rounded to the nearest integer. ^b^Approximate estimation

## Population and enrolment

The target population was based on the Safe Step observational study conducted in 2019–2021 in Östersund municipality, a middle-sized municipality in Sweden with approximately 64,000 inhabitants, of which 16% were 70 years and older (Statistics Sweden 2023a). A description of the Safe Step intervention (Pettersson et al. 2020) and eligibility criteria are reported in detail elsewhere (Bajraktari et al. 2022). The intervention was designed to include community-dwelling people 70 years or older with potential fall risk, e.g. impaired balance, and digital skills such as using email or smart technology regularly. Using an online inclusion process, participants self-assessed whether they met the eligibility criteria and enrolled by providing their email address through the project’s website and subsequently answering the baseline questionnaire. A total of 173 participants were included in the study. The participants’ mean age was 76 years, and 70% were women (Bajraktari et al. 2022).

## Intervention

The Safe Step intervention consisted of a fully self-managed exercise programme, meaning it was provided via a mobile application without the involvement of healthcare professionals. It also included monthly educational videos on fall prevention sent via email and optional supportive strategies (Bajraktari et al. 2022). Exercises (balance, strength, and gait) in the programme were shown in short videos, and participants were guided to compose their individualized programme by choosing ten exercises that were challenging but not too hard (Pettersson et al. 2020). The monthly educational videos sent by mail included short information on various topics related to healthy ageing and falls prevention. Supportive strategies for exercising with the application were available in the format of introductory drop-in meetings, group exercise sessions, and technical support (Bajraktari et al. 2022).

## Model structure

A Markov model was chosen due to the extended time horizon of 12 years. The states included in the model were: *At risk for fall* (state A), *Minor consequences* after a fall (state B), *Major consequences* after a fall (state C), *Special housing* after a fall (state D), and *Death* (state E). Modelling was utilised to capture long-term consequences of a fall, including the impact on quality-adjusted life years (QALYs) and societal costs. The model was based on scientific literature on falls burden and consequences and further validated by members within the research group with extensive experience from research on falls. Definitions for each state and possible transitions are illustrated in Fig. 1.

## Transition probabilities

Transition probabilities (TPs) reflect annual probabilities to move between states in the model (Table 1). As an example, a person in state A can transition to any other state (e.g. 7% probability to transition to B) or remain in state A (72% probability). The cycle in the model was one year; at the beginning of the first year, all participants were assumed to start in state A. For state A, all TPs from state A to the other states in the model are based on statistics for the Swedish population of age 65 + . The TPs to state B and C represent statistics on fall-related mild (e.g. wrist fracture) and severe (e.g. hip fracture) injuries (Borgström et al. 2006). The TP to state D represents the probability to move to long-term care (Socialstyrelsen 2022b). The TP to state E during the first year represents the age-specific mortality rate for people aged 76 years (Socialstyrelsen 2023), the mean age in the Safe Step observational trial. For subsequent years, the TPs from A to E were adjusted for the increased age-related mortality risk.

For state B, TPs to state C represent likelihood for a subsequent hip fracture after having experienced a wrist fracture (Crandall et al. 2015). The TP to remain in B represents the probability for sustaining an upper extremity fracture subsequent to a prior occurrence (Crandall et al. 2015). TPs to the states D and E are assumed to be the same as for those in state A. Thus, from the second years and onwards, the TPs from B to E were adjusted for the increased age-related mortality risk.

For state C, TPs represent findings on gradual recovery (transition to B) or a combination on little and no recovery (remain in C) after a serious fall-related injury (Gill et al. 2013). TP to D represents the probability to move to long-term care following a hip fracture (Dyer et al. 2016). TP to E represents mortality rate within a year following a hip fracture in the Swedish population of 60 years and older (Meyer et al. 2021). Finally, TP from C to A represents those that remain in C after removing those that transition to the other states.

For state D, it was assumed that it is either possible to remain in state D or to move to state E, probability to recovery to other states (C, B, A) was considered negligible. TP to state E represents the death rate one year after admission in long-term care (Folkhälsomyndigheten 2023; Socialstyrelsen 2021).

## Quality of life

We used quality-adjusted life year (QALYs), to capture the impact of a fall on a person’s health-related quality of life. A fall can affect function by limiting participation in everyday activities which in turn can affect quality of life (Borgström et al. 2006). Thus, QALYs were assigned to each state, see Table 2.

For state A, the health-related quality of life score was based on baseline data from the Safe Step observational study (Bajraktari et al. 2022).

For states B and C, health-related quality of life scores were based on a Swedish observational study assessing quality of life and costs in osteoporosis-related wrist, hip, and vertebra fractures in older people one year after the fracture (Borgström et al. 2006). Wrist and hip fracture-related consequences (decrements in QALY) were assumed to represent states B and C, respectively.

For state D, we used health-related quality of life score approximations for a state of total dependency in instrumental activities of daily living (IADL) and personal activities of daily living (PADL) resulting in the need for long-term care in special housing. The health-related quality of life score was extracted from a Swedish cost-effectiveness study, modelling an intervention to reduce bathing disability in older people (Zingmark et al. 2016), which made their estimation upon findings on decrements in QALYs due to major loss of independence (Andersen et al. 2004) and move to long-term care (Honkanen et al. 2006).

## Societal costs

A societal perspective was utilized to evaluate costs. Consequences of a fall on societal costs were described in terms of need for care or assistance in various formats, provided either by the region (e.g. emergency care, specialised care, primary care), the municipality (e.g. homecare service, long-term residential care facility), or family (informal care)**.** Homecare services in Sweden includes help with activities of daily living (ADL), which a person cannot complete themselves. Homecare services are allocated to an individual after a needs assessment procedure (Socialtjänstlag [SFS], 2001). Costs were assigned for states B, C, and D. No costs were assigned for states A and E.

Costs for states B, C, and D were based on cost estimates for major and minor fall-related injuries, as reported in a recent report on cost-effectiveness of evidence-based fall preventive interventions issued by the Swedish National Board of Health and Welfare (Socialstyrelsen 2022a). State B included direct costs paid by the region. No indirect costs were accounted for in this state due to the reported low severity of these type of falls not needing extended care or help which would affect informal care or care provided by the municipality (Borgström et al. 2006). State C included direct cost paid by the region and municipality and indirect costs (informal care). State D included costs for special accommodation in long-term care. Costs are given in Euro (€) and adjusted to December 2021 values (Statistics Sweden, 2022a) (Table 2).

## Intervention effect

The intervention effect was based on a meta-analysis focused on fall preventive exercise interventions for community-dwelling people 60 years or older (Sherrington et al. 2020). The mean age of participants in the 116 included RCTs was 76 years, and 74% of participants were women. Intervention effects were evaluated for exercise intervention in general versus control but also for specific types of exercises, e.g. effect of balance and functional exercise versus control (Sherrington et al. 2020). The intervention effect applied in this model refers to the effect reported for programmes involving balance and functional exercise interventions. The intervention effect was RaR (rate ratio) 0.76, meaning a 24% reduction in the rate of falls in the intervention group (Sherrington et al. 2020). In the model, we implemented the intervention effect as a reduced risk for transitions from state A to states B and C, as well as from state B to C. The intervention was assumed to have an effect only for the first year and not for the second to twelfth years.

## Intervention cost

Intervention costs were based on actual costs from the Safe Step observational study including costs for recruitment, supportive strategies, and an estimate of the annual maintenance cost for the Safe Step application (Table 3). Recruitment strategies and additional supportive strategies were implemented and financed by the municipality to optimize reach (Bajraktari et al. 2022). The maintenance cost for the Safe Step application was financed within the larger research project.

The recruitment costs included: a brochure (including actual costs for printing and posting costs) distributed to persons 70 years and older in the municipality, bus advertisement (actual costs), oral presentations at voluntary organizations for older people (Bajraktari et al. 2022), Table 3. Costs for oral presentations were estimated based on the direct labour hours put on presentations which than were multiplied with an average market wage. Optimally, a fall preventive intervention would be delivered by trained professionals, such as physiotherapists or occupational therapists. Therefore, we estimated that a salary of 25.6 €/h would be an appropriate gross wage per hour based on the average salaries for physiotherapists and occupational therapists in 2021 (Statistics Sweden 2022b). Time spent on planning and coordinating the presentations was not accounted for in the cost calculation of recruitment strategies.

The supportive strategies included costs for group exercise sessions and salaries for personnel involved in introductory drop-in meetings and technical support. The annual maintenance and operational costs for the application were estimated based upon contact with developers of the application. The average cost per participant was €121, calculated as the total intervention cost divided by the total number of participants in the Safe Step observational study (Bajraktari et al. 2022). Of the total intervention cost per participant, recruitment costs represented €52, supportive strategies €42, and application-related costs €27 (Table 3).

## Analysis

Microsoft® Excel version 2010 was used to analyse the Markov model over a time-period of 12 years, corresponding with the remaining life expectancy at the age of 76 (Statistics Sweden 2023b). In the base-case analysis, we compared accumulated QALYs and societal costs for the intervention in relation to no intervention. In the analysis, we applied half-cycle correction. We discounted future costs and QALYs by 3% annually after the first year (The Dental and Pharmaceutical Benefits Agency, 2017). The difference in costs was divided by the difference in benefits (QALYs gained) to estimate the incremental cost-effectiveness ratio (ICER). No health economic analysis plan was published.

## Sensitivity analysis

Deterministic sensitivity analysis was employed to assess parameter uncertainty regarding intervention effect and intervention cost (Table 4). Three different scenarios were evaluated.

**Table 4:** Input parameters in deterministic sensitivity analysis and base-case analysis

Parameters (Per person)	Base case	Scenario 1	Scenario 2	Scenario 3
Intervention cost^a^				
Recruitment cost	52	Base case	78	Base case
Supportive strategies costs	42	Base case	Base case	Base case
Maintenance costs	27	Base case	Base case	Base case
Intervention effects	0.76	0.84	Base case	0.98

^a^Costs are reported in Euros (€) and rounded to the nearest integer

In scenario 1, we assumed a lower intervention effect of 0.84. This treatment effect is reported in a randomised controlled trial with a two-year follow-up evaluating a digital fall prevention exercise programme, the StandingTall programme (Delbaere et al. 2021). The StandingTall programme is administered via an app and evaluated among community-dwelling people, 70 years or older, in Australia (n = 503) (Delbaere et al. 2021). In scenario 2, we assumed 50% higher recruitment costs simulating that a larger campaign had been implemented. In scenario 3, we assumed a conservative intervention effect of 0.98, representing the higher level of the confidence interval in the StandingTall study (Delbaere et al. 2021), simulating a conservative scenario where the intervention would only decrease the chance for a fall with two per cent.

## Results

In the base-case analysis, the Safe Step intervention was less expensive and more effective than no intervention, thereby the intervention dominated no intervention. When considering the total population of 173 participants over the 12 years for which the intervention was evaluated, 10.04 QALYs were gained (0.058 QALY per participant), and societal costs were reduced with €144 282 (€834 per participant) Table 5. In a one-year perspective, participants in the intervention group remained in more favourable states in comparison with no intervention; eight falls with either minor or major consequences would be prevented.

**Table 5:** Quality-adjusted life years (QALYs) and costs for base-case and sensitivity analyses

	No intervention (n = 173)		Intervention (n = 173)			Incremental QALYs/ participant	Incremental costs/ participant
	QALYs	Costs		QALYs	Costs		
Base case	4.490	104 370		4.548	103 535	0.058	−834
Sensitivity analysis							
Scenario 1	4.490	104 370		4.529	103 852	0.039	−517
Scenario 2	4.490	104 370		4.548	103 560	0.062	−809
Scenario 3	4.490	104 370		4.495	104 406	0.005	37

The table shows accumulated QALYs (quality-adjusted life years) and costs for the intervention and the comparator (no intervention) as well as incremental QALYS and incremental costs over 12 years. All values are reported per participant. All three scenarios are based on the base-case analysis but include changes in a specific parameter as described below. Scenario 1: lower intervention effect in comparison with base case (StandingTall fall preventive digital intervention treatment effect) (Delbaere et al. 2021); Scenario 2: increased recruitment cost with 50%; Scenario 3: intervention effect assumed to be conservative, representing the higher level of confidence interval of the StandingTall intervention (Delbaere et al. 2021)

In the sensitivity analysis (scenarios 1 and 2), the Safe Step intervention resulted in health gains and lower costs than no intervention, hence dominated no intervention. Assuming a lower intervention effect in scenario 1, 6.70 QALYs were gained in total for all 173 participants (0.039 QALY per participant) and societal costs were reduced by €89 519 (€517 per participant). Assuming a 50% larger recruitment cost in scenario 2, the same number of QALYs were gained, whereas the societal costs were reduced in total by €139 997 (€809 per participant). Assuming a conservative intervention effect in scenario 3, 0.837 QALYs were gained in total for all participants (0.005 per participant), whereas societal costs increased in total with €6 375 (€37 per participant) resulting in an ICER of €7 616 per QALY gained, see Table 5 for data on QALYs and costs per participant.

## Discussion

The results from this study based on a Markov model indicate that the digital intervention Safe Step is a cost-effective approach to prevent falls among community-dwelling older people at risk for falling. Results from our base-case scenario as well as two sensitivity analyses indicate that the Safe Step intervention is both cost-saving and result in health gains in comparison with no intervention. Additionally, the results from a third sensitivity analysis show that even with a very conservative estimate of the intervention effect, the intervention has the potential to result in QALY gains and be cost-effective even though societal cost were higher in comparison with no intervention.

The interpretation of cost-effectiveness depends on established threshold values that represent a decision-maker’s willingness to pay for a QALY. These thresholds are not fixed and can vary from country to country (Culyer 2016). An analysis conducted by the Swedish National Board of Health and Welfare suggests that costs up to €100,000 per QALY gained would be acceptable (Socialstyrelsen 2022a). Therefore, an ICER of €7 406 per QALY gained (scenario 3) lies within the range of what is considered acceptable in Sweden (Socialstyrelsen 2022a). It is also important to notice that even if fall preventive exercise does not directly reduce falls it can have positive effects in falls risk factors, such as improved balance or leg strength, positive effects that are not directly reflected in instruments developed to value health-related quality of life, e.g. the EQ-5D (Bjerk et al. 2019).

Comparing our results with other similar studies is rather difficult considering the scarce and scattered cost-effectiveness studies related to fall prevention exercise in community-dwelling older people in general (Olij et al. 2018; Winser et al. 2020; Davis et al. 2010) and specifically digital ones (Ambrens et al. 2022). Most cost-effectiveness studies, on fall preventive exercise, focus on evaluating traditional forms of fall prevention including supervision by a health care professional such as a physiotherapist either in a group or individual format. With an increasing population of older people and difficulty to recruit and retain healthcare personnel (Statistics Sweden 2021), it will become eminent to find innovative approaches that can facilitate a more efficient allocation of resources. Based on our results, we conclude that the Safe Step application is a cost-effective intervention that seem feasible to implement to reach older people at risk of falling who are familiar with technology (Bajraktari et al. 2022).

In line with our results, findings from the Swedish National Board of Health and Welfare reporting on health economic evaluations of different evidence-based fall preventive programmes found exercise interventions to be cost-effective and cost-saving in community-dwelling older people 65 years or older (Socialstyrelsen 2022a). Findings from systematic reviews of economic evaluations of falls prevention intervention vary depending on the target group, type of intervention, format of delivery and threshold applied for comparison. A systematic review of economic evaluations of fall preventive interventions indicated that, at a threshold of $50 000/QALY, about two-thirds of all included interventions were deemed cost-effective (Olij et al. 2018). However, whereas home assessment programmes were the most cost-effective type of intervention for community-dwelling older adults, medication adjustment was the most cost-effective intervention for older people living in special housing. Another systematic review that evaluated cost-effectiveness of fall preventive exercise interventions suggested that exercise interventions are cost-effective independent of the delivery format (Winser et al. 2020). A more recent review including 21 economic evaluations of exercise interventions found most interventions to be cost-effective at a threshold of $100 000/QALY, however, unsupervised exercise interventions resulted in higher ICERs, and therefore were considered low value for money (Pinheiro et al. 2022). The conclusion that unsupervised exercise interventions were less cost-effective was based on studies, all evaluating the Otago exercise programme, where half of the studies did not find the interventions evaluated to be cost-effective. In contrast to previous studies on fall preventive exercise, Safe Step is delivered in a digital format. A more similar intervention to Safe Step, the StandingTall intervention recently reported results on cost-effectiveness of a home-based exercise programme aimed at reducing falls in community-dwelling older people. The StandingTall intervention was mainly digital and included fall prevention exercise to be performed at home in combination with two home visits and occasional telephone calls to support adherence to the exercise. Results from the cost-effectiveness analysis of the StandingTall intervention reported an ICER per QALY gained of $58 039, which was deemed cost-effective in relation to a threshold of $60 000/QALY (Ambrens et al. 2022). In contrast to StandingTall, Safe Step dominated no intervention and was cost-saving in all analyses except for the sensitivity analysis with the most conservative estimate of intervention effect.

In contrast to other cost-effectiveness studies on falls prevention, the total costs per participant were lower (Davis et al. 2010, 2020; Ambrens et al. 2022). The Safe Step intervention was developed to be fully self-managed with minimal individual contact, and therefore, large costs related to e.g. supervision by professionals can be excluded. Nevertheless, we included recruitment- and support-related costs implemented with the goal to improve reach of the intervention. Considering advanced age as one of the risk factors for digital exclusion, digital interventions complimented with additional support could potentially be effective in the attempt to improve reach. However, the supportive strategies applied in this study did not show to be as effective we anticipated them to be based on the low number of participants attending (Bajraktari et al. 2022), which could indicate that those enrolled in the study did not need such support. Nevertheless, the cost for supportive strategies was substantial €42 per participant or 35% of the intervention costs. Thus, the cost of such support needs to be considered in relation to potential benefits both in future research and in practice.

In this study, recruitment costs represented about 45% of the total intervention costs. Reaching the target population is pivotal for the success and cost-effectiveness of community-level interventions (Culyer 2016). In a previous study on Safe Step, we reached 4.7% of the estimated target population (Bajraktari et al. 2022). With a higher reach, intervention costs would have been distributed over larger number of persons and thereby reduced. However, whereas the intervention costs, including recruitment strategies, are relatively small in relation to the fall-related costs for states B, C and D, the main benefit of successful recruitment strategies would be if reach was further improved and larger numbers of people in the target population accessed Safe Step and thereby reduced their risks for fall-related consequences. Whereas the actual intervention effect of Safe Step remains to be established, such insights could be an incitement for decision-makers to allocate more resources for prevention, including strategies to increase reach, thereby increasing the impact of an intervention at the population level (Winser et al. 2020; Culyer 2016). Whereas less than 50% of health economic evaluation studies on fall prevention exercise for community-dwelling older people reported recruitment-related costs (Pinheiro et al. 2022), we consider recruitment-related actions and costs an issue not to be underestimated.

## Limitations

Some limitations should be considered when interpreting the results of this study. In our model, we did not account for the increased risk of falling with advanced age and neither for the higher likelihood of falls in women in comparison with men (Montero-Odasso et al. 2022). To model the intervention, we used the intervention effect reported in a systematic review on traditional exercise interventions since, to this date, no available intervention effect is available on Safe Step. To account for this limitation in a sensitivity analysis, we reduced efficacy of the intervention at first by using the mean intervention effect level reported by the StandingTall intervention and second, we applied a very conservative estimate to avoid an overestimation of cost-effectiveness: the upper level of the confidence interval of the StandingTall intervention (Delbaere et al. 2021). Since the intervention effect has a strong influence on the cost-effectiveness of the intervention, the precision of our model can likely be enhanced when data on intervention effects of digital falls preventions interventions including the Safe Step intervention is available.

Whereas transition probabilities were derived primarily from Swedish studies, a limitation is that we for some transitions could not identify Swedish studies and therefore used data from international studies to estimate transition probabilities. Further, transition probabilities are not directly related to the age of 76 + , and transitions are extracted for different age groups, although none of them for persons under 60 years of age.

Costs incurred in conjunction with the planning of the intervention and involving staff working at the municipality, e.g. communication officers, were not included in the intervention cost, and thereby, intervention costs might have been underestimated. Additionally, QoL scores for states B and C might have been underestimated since they are based on a study assessing quality of life in persons with osteoporosis-related fractures who in general report lower QoL scores than those in the general population (Patel et al. 2013; Beaudart et al. 2017).

With regard to model structure, the choice of levels was based on previous research, but validation was limited to thorough discussions in the research group rather than validation by external researchers or experts. However, the results from our study are similar to those presented by the Swedish National Board for Health and Welfare (2022a; b) which to some extent support cross-validation.

With regard to the sensitivity analyses, we choose a stepwise approach, instead of a probabilistic approach. Whereas this choice facilitates interpretability of how the results were affected by changes in key parameters, we acknowledge that all parameters in a model can be subject to uncertainty. Once the intervention effect of Safe Step has been established, a probabilistic sensitivity analysis can provide additional information about the probability of the intervention being cost-effective in relation to established thresholds.

## Conclusions

The results indicate that Safe Step is a cost-saving intervention in preventing falls in community-dwelling older people. Even in a sensitivity analysis based on a very low intervention effect, the intervention was clearly cost-effective in relation to a threshold of €100,000 QALY/gained. The intervention was still cost-saving even when recruitment costs were raised with 50%, indicating that intervention effect rather than intervention cost influence the magnitude of the cost-effectiveness. Future studies on the effectiveness of fall preventive digital interventions will contribute in precising effect estimates and enhance the validity of cost-effectiveness results.

## Data Availability

Upon reasonable request, the datasets utilized in the present study can be provided
